# Methanation of CO_2_ over Ruthenium Supported on Alkali-Modified Silicalite-1 Catalysts

**DOI:** 10.3390/molecules28176376

**Published:** 2023-08-31

**Authors:** Michał Zieliński, Ewa Janiszewska, Adrianna Drewniak, Mariusz Pietrowski

**Affiliations:** Faculty of Chemistry, Adam Mickiewicz University, Uniwersytetu Poznańskiego 8, 61-614 Poznań, Poland; adrdre@st.amu.edu.pl (A.D.); mariop@amu.edu.pl (M.P.)

**Keywords:** ruthenium catalyst, modified silicalite-1, surface basicity, CO_2_ methanation

## Abstract

This study focuses on the catalytic properties of ruthenium catalysts supported on modified silicalite-1 (with an MFI structure). By post-synthesis modification of silicalite-1 with solutions of alkali metal compound, a novel and cost-effective method was discovered to create basic centers on the surface of silicalite-1 supports. The modification not only affected the basicity of the supports but also their porosity. The influence of the type of alkali solution (KOH or NaOH) and its concentration (0.1 M or 1.0 M) on both the basicity and porosity was investigated. The modified silicalite-1 materials were employed as supports for ruthenium catalysts (1 wt.% Ru) and evaluated for their CO_2_ methanation activity. The results were compared with the hydrogenation performance of ruthenium catalysts supported on unmodified silicalite-1. Characterization of the supports and catalysts was conducted using techniques such as BET, XRD, FT-IR, ICP-OES, TPR-H_2_, H_2_ chemisorption, TPD-CO_2_, SEM, and TEM. Remarkably, the catalytic activity of ruthenium supported on silicalite-1 treated with 1.0 M NaOH (exhibiting selectivity toward methane above 90% in a reaction temperature range of 250–450 °C) outperformed both unmodified and KOH-modified silicalite-1 supported Ru catalysts.

## 1. Introduction

The expanding human activities and continuous advancement of industrial technologies greatly contribute to ongoing climate changes, with the primary cause being the increasing concentration of carbon dioxide in the atmosphere. Carbon dioxide, as a greenhouse gas, has a significant impact on amplifying the greenhouse effect. Therefore, various attempts have been made to utilize CO_2_ generated from industrial processes to minimize its emissions into the atmosphere and improve the state of our environment. Significant efforts are being made to replace conventional fossil fuels with renewable energy sources such as wind or solar energy.

One of the main challenges associated with the growth of renewable energy production is intermittency, which requires balancing electricity generation and/or storing energy surpluses [1]. On the other hand, CO_2_ emissions result in the wastage of large amounts of carbon, which is a building block for fossil fuels and petrochemical products. The most commonly utilized technology is carbon capture and storage (CCS), which involves capturing CO_2_, transporting it, and storing it underground. Alternatively, captured CO_2_ can be utilized and transformed into fuels and chemicals, such as dry methane reforming for synthetic gas production or CO_2_ hydrogenation into methane, methanol, or higher alcohols.

The CO_2_ methanation reaction was discovered by Sabatier and Senderens back in the 19th century [2]. However, currently, it is primarily utilized in research in the sustainable energy industry and is commonly found alongside hydrogen production processes (e.g., water electrolysis) in most projects. The process is known as power-to-gas technology. In power-to-gas technology, excess renewable electricity is used to produce hydrogen through PEM (*Proton Exchange Membrane*) electrolysis technology, which is then reacted with CO_2_ to undergo a chemical transformation into methane. The hydrogenation of CO_2_ to produce methane, known as the Sabatier reaction, is an exothermic reaction (1):

CO_2_ + 4H_2_ → CH_4_ + 2H_2_O         ΔH_298K_ = −252.9 kJ·mol^−1^ (1)

and is thermodynamically favorable (ΔG_298K_ = −130.8 kJ·mol^−1^). Indeed, other reactions can occur, as the weakly endothermic reverse water gas shift—RWGS (2):

H_2_ + CO_2_ → CO + H_2_O          ΔH_298K_ = 41.2 kJ·mol^−1^ (2)

or reactions involving CO (3) and (4):

CO + 3H_2_ → 4CH_4_ + H_2_O         ΔH_298K_ = 206.1 kJ·mol^−1^ (3)

2CO → C + CO_2_        ΔH_298K_ = 172.4 kJ·mol^−1^ (4)

Methane, as an energy carrier, can be stored for later use at a different location or time, or injected into the existing natural gas grid. This allows for the efficient storage and utilization of renewable energy, providing flexibility and balancing the intermittent nature of renewable sources.

In recent years, research has intensified regarding the acquisition of a catalyst that exhibits high activity and selectivity in this reaction, while also being resistant to high temperatures. Besides the active phase, the support material is an important component of an active and selective catalyst, and both the active phase and its support play crucial roles in catalytic process. Metal–support interactions can modify the catalytic properties of the metallic phase, thus emphasizing the significance of the support’s nature in the reaction pathway [3,4]. Catalysts based on Ru, Rh, and Pd supported on different oxides (Al_2_O_3_, TiO_2_, ZrO_2_, CeO_2_, or MgO) have exhibited excellent catalytic properties in CO_2_ methanation [5,6,7,8], among which Ru is the most active component at low temperature [9,10,11,12].

Furthermore, it has been shown that the catalytic activity and selectivity of CO_2_ hydrogenation catalysts can be improved by doping the supports with various additives, such as alkali metals (Na, K, Li, Cs) [4,13,14,15,16,17,18,19] and alkaline earth (Ca, Ba, Sr, Mg) [15,20,21], as well as lanthanides (La, Ce) [15].

Within the aforementioned context, the aim of this study was to use silica support with an MFI structure (silicalite-1) with varying basicity as a support for a ruthenium catalyst. The basic centers on the surface of silicalite-1 supports were created by post-synthesis modification of silicalite-1 with solutions of alkali metal hydroxides (KOH or NaOH) of different concentrations (0.1 M or 1 M). The impact of support basicity on the efficiency of the resulting ruthenium catalysts in CO_2_ hydrogenation to methane was investigated. Considering the influence of support texture and basic functionality on catalyst activity, the supports and catalysts were characterized using techniques such as low-temperature nitrogen adsorption/desorption (BET), X-ray powder diffraction (XRD), Fourier-transform infrared spectroscopy (FTIR), temperature-programmed reduction with hydrogen (TPR-H_2_), inductively coupled plasma optical emission spectroscopy (ICP-OES), H_2_ chemisorption, temperature-programmed desorption of carbon dioxide (TPD-CO_2_), scanning electron microscopy (SEM), and transmission electron microscopy (TEM). The catalytic activity of ruthenium catalysts supported on unmodified and modified silicalite-1 was compared. The effect of catalyst texture and basicity on the activity of ruthenium catalysts in the hydrogenation of CO_2_ to produce methane was evaluated. To the best of our knowledge, there are no available data on such modifications of silicalite-1 followed by their application as support materials for ruthenium catalysts for hydrogenation processes.

## 2. Results and Discussion

**Figure 1:**
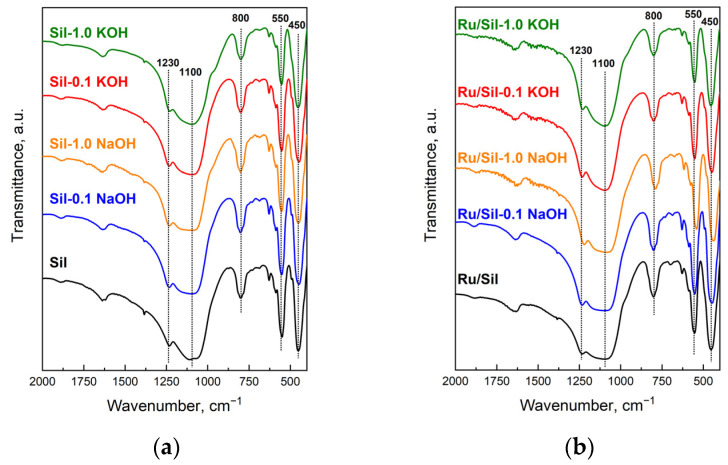
FTIR spectra of indicated supports (**a**) and catalysts (**b**).

The FTIR spectra (Figure 1) of the initial material (Sil) and modified silicalite-1 samples show bands typical for siliceous materials and which are insensitive to the structure (1230, 1100, 800, and 450 cm^−1^), and a band originating from the vibration of the double five-ring of the MFI framework [22]. The presence of a band at 550 cm^−1^ in the spectra of modified samples suggests that the MFI structure was maintained after the alkaline treatment of silicalite-1. The high intensity of this band indicates good crystallinity of the modified samples [23]. Moreover, the ratio of the intensities of bands at 450 and 550 cm^−1^ for modified samples enables the calculation of their crystallinity by comparison to the ratio of these bands in unmodified silicalite-1, whose crystallinity is regarded as 100% (Table 1).

The results indicate a decrease in the crystallinity of modified silicalite-1 samples in comparison to the unmodified Sil, due to the extraction of silicon species from the framework with the formation of the defects containing sodium or potassium ions. The increase in the concentration of the solution used for modification caused a higher decrease in crystallinity regardless of the used alkali compound. It is caused by the formation of a higher number of defects in the structure of silicalite-1 during modification with solutions of higher concentration. Comparing the crystallinity of the samples modified with NaOH to the crystallinity of the samples modified with KOH (for the same concentration), a higher loss of crystallinity was observed for samples modified with KOH solutions. The crystallinity of Sil-1.0 KOH was only 66%, whereas the crystallinity of Sil-1.0 NaOH was 73%. This indicates a better tolerance for NaOH treatment than for KOH modification. The introduction of the ruthenium phase on the unmodified and modified silicalite-1 caused a further decrease in the crystallinity of the used supports. The highest loss was observed for unmodified silicalite-1 (14%). For catalysts with alkali-modified supports, a higher loss of crystallinity was observed for supports modified with an alkali solution of lower concentration, regardless of the alkali compounds used. The source of ruthenium used was ruthenium chloride and, in our previous work, we have shown that modification of silicalite-1 with NH_4_Cl and NH_4_OH influences the structure, causing the decrease in crystallinity [22]. This suggests that, in the conditions of impregnation used, chlorine ions present in RuCl_3_ act as a structure modifier, similarly to chlorine in the procedure of modification used by NH_4_Cl. On the other hand, XRD data presented below show the formation of NaCl or KCl during impregnation on supports modified with 1.0 M XOH (X- K or Na). This indicates that chlorine from RuCl_3_ reacts with sodium and potassium ions present in defects formed in the framework of silicalite-1. It seems that, in this case, Na^+^ and K^+^ protect the structure from modification by Cl^−^. The protection is higher for supports with a higher amount of alkali ions, which is equivalent to a higher amount of defects. The higher concentration of defects is present in supports modified with 1.0 M XOH, which explains their lower sensitivity to the action of chlorine during the impregnation process.

**Figure 2:**
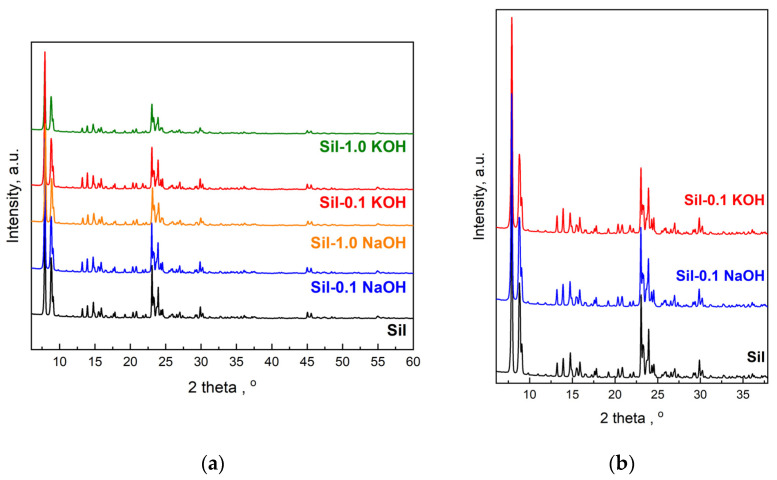
Comparison of XRD patterns of pristine and modified silicalite-1 samples (**a**) and patterns of samples modified with solutions of different alkali compounds of the same concentration (**b**).

Figure 2 shows the powder X-ray diffraction (XRD) patterns of the initial silicalite-1 and the modified samples. It can be seen clearly that all supports exhibit well-resolved MFI structure peaks at 2Θ = 6–60° [24,25]. The diffraction peaks of silicalite-1 samples modified with a solution of KOH or NaOH are slightly weaker than those of the initial silicalite-1. It indicates that the modification with an alkali solution caused a decrease in the crystallinity of silicalite-1. The decrease in crystallinity increases with the concentration of the alkali solution used for modification (Figure 2a). Comparison of the intensity of reflection for samples modified with NaOH and KOH of the same concentration indicates a higher decrease in crystallinity for samples modified with KOH solutions (Figure 2b). The obtained results of XRD analysis are in line with the FTIR data.

**Figure 3:**
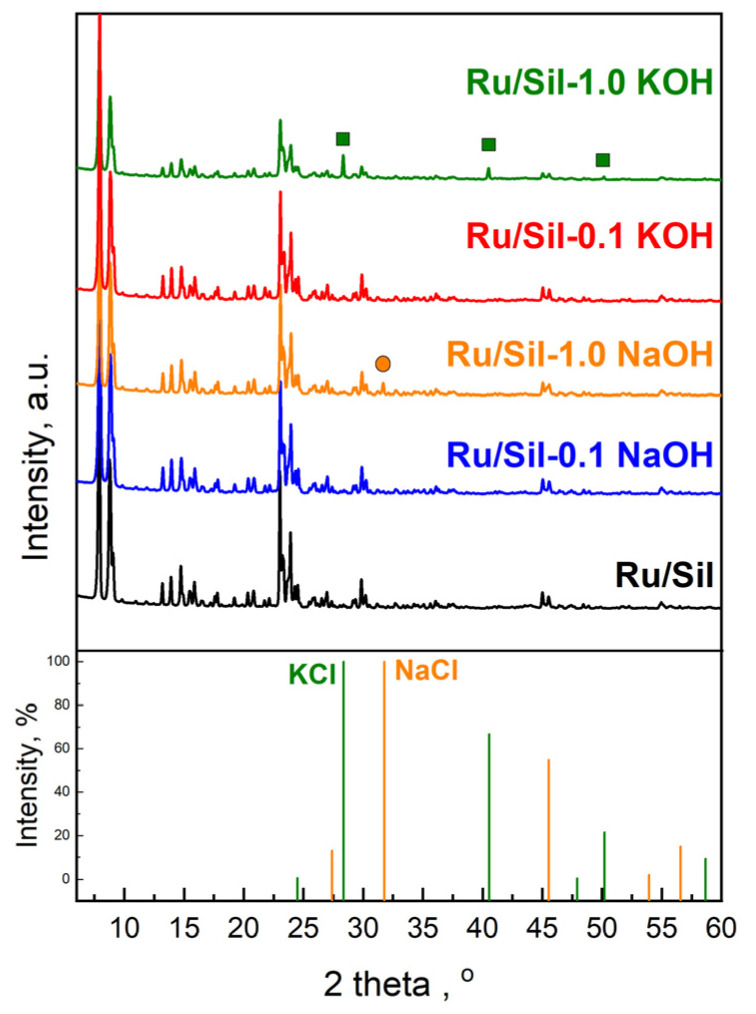
Comparison of XRD patterns of indicated catalyst, and NaCl and KCl.

The crystallinity of the catalysts is almost comparable to that of the supports (Figure 3). It indicates that the conditions of impregnation used are mild enough and do not affect the structure of the supports. Characteristic peaks of metallic Ru species (2θ = 44.37°) were not observed due to the low ruthenium content (1 wt.%—determined by the ICP-OES method) as well as the high dispersion of ruthenium species on the supports. Similarly, the absence of reflections from ruthenium, at low active phase loadings, was observed by the authors of the paper [26]. Only for catalysts supported on samples modified with 1.0 M potassium and sodium solutions, additional reflection at 2θ = 28.314°, 40.472°, and 50.127° attributed to KCl crystals (JCPDS Card number 41-1476), and 2θ = 31.693° attributed to NaCl crystals (JCPDS Card number 72-1668), were observed, respectively (Figure 3). This indicates the reaction of alkali ions (Na^+^ and K^+^) occurring in the defects with chloride ions present in the ruthenium precursor. The above-mentioned reflections are not observed in patterns of catalysts supported on Sil-0.1 NaOH and Sil-0.1 KOH due to low concentrations of sodium and potassium in these supports.

**Figure 4:**
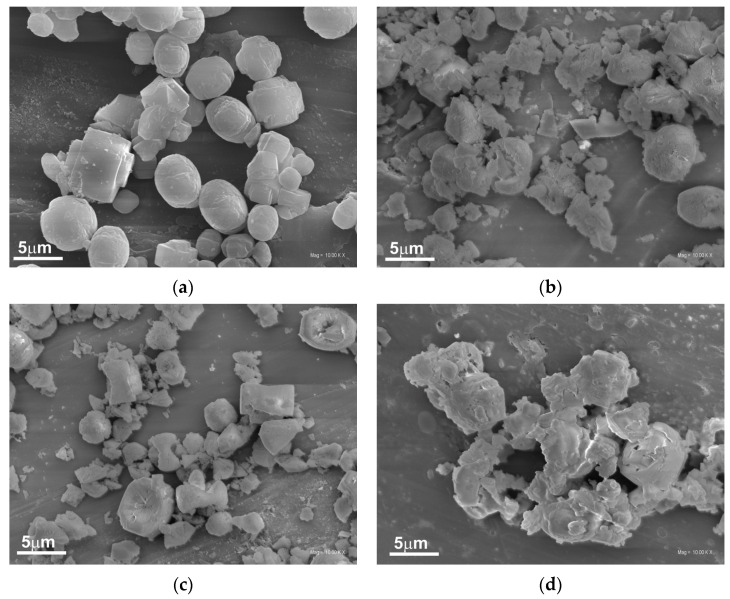
SEM images of the supports: Sil (**a**), Sil-0.1 KOH (**b**), Sil-0.1 NaOH (**c**), and Sil-1.0 KOH (**d**).

The morphology of the original and the alkali-treated silicalite-1 was studied by scanning and transmission electron microscopies. SEM images show the influence of the type and concentration of modifiers on the morphology of the obtained supports (Figure 4a–d). The unmodified silicalite-1 (Figure 4a) possesses well-formed, separated twin crystals typical for MFI materials with a smooth surface [27].

However, the modification with 0.1 M concentration of alkali solution caused the damage of the crystals with visible detached and/or amorphous segments, as shown in Figure 4b,c. The damage of the crystals is more severe for samples modified with a higher concentration of alkali solution, and, in this case, the damaged crystals are connected in larger agglomerates (Figure 4d).

**Figure 5:**
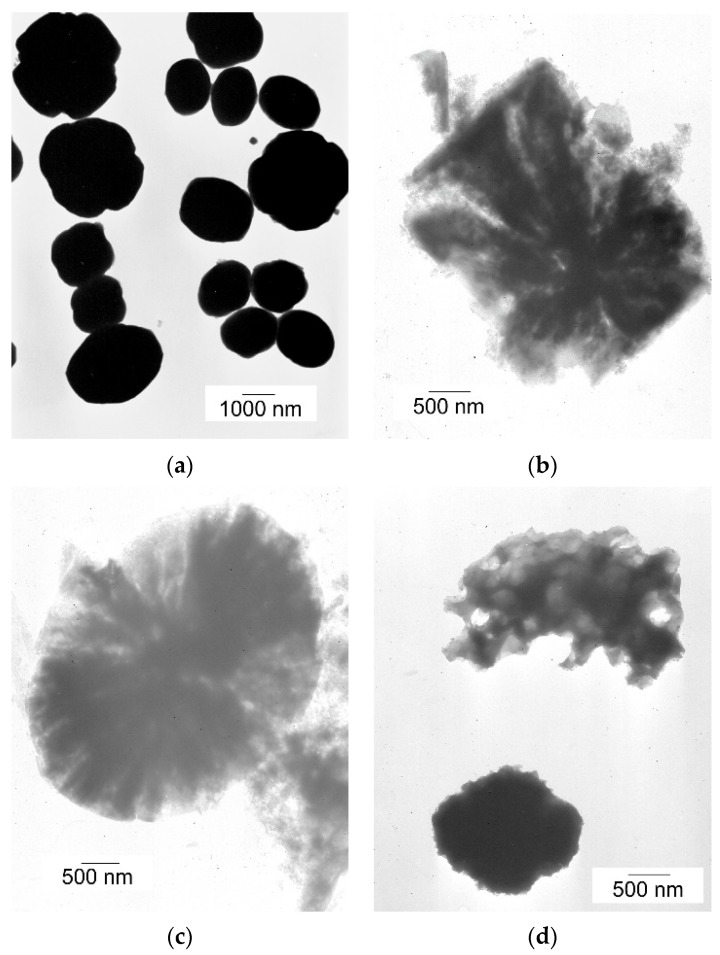
TEM images of unmodified (**a**) and modified with 0.1 M NaOH (**b**), 0.1 M KOH (**c**), and 1.0 M KOH (**d**) supports.

The alteration in the morphology of the crystals and the formation of additional pores after alkali modification can also be observed in the TEM images of the modified samples (Figure 5). No porosity is visible in the crystals of the initial silicalite-1 (Figure 5a), whereas the crystals of modified samples exhibit etched irregular pores. The crystals of supports modified with a 0.1 M alkali solutions (Figure 5b,c) show additional pores in the form of the channels, and the sample treated with KOH displays more open structures with larger pores, which is consistent with the data from XRD and FTIR analyses. The TEM image of the support treated with 1.0 M KOH (Figure 5d) shows the more pronounced alterations in the crystals, and the additional pores are bigger compared to the pores formed after treatment with 0.1 M solutions, and they look like holes.

The textural properties of the supports and catalysts were characterized by the low-temperature nitrogen adsorption–desorption measurements. The modification of silicalite-1 with low-concentration alkali solutions resulted in an increase in specific surface area (SSA) and total pore volume, whereas modification with a more highly concentrated solution caused a decrease in these values (Table 1). The alkaline treatment of silicalite-1 also led to an increase in mesoporosity (mesopore surface area (S_ext_) and volume (V_meso_)), accompanied by a loss of microporosity (V_micro_). The exception is sample Sil-1.0 KOH, which shows a decrease in mesoporosity compared to the mesoporosity of the unmodified sample. This could be a result of higher degradation of the structure by modification with 1.0 M KOH, in comparison to the influence of other solutions on the structure of silicalite-1. The observed decrease in micropore volume for all modified samples is assigned to a loss of crystallinity, which is in agreement with XRD and FTIR data [28]. The introduction of the ruthenium phase on the surface of the supports practically did not change the specific surface area and total pore volume. However, differences in microporosity and mesoporosity between catalysts and supports were observed. A decrease in microporosity was observed, accompanied by an increase in mesopores, as a result of further deterioration of the silicalite-1 structure of the supports by chloride ions during the impregnation procedure. The impregnation with the ruthenium phase led to the highest drop in micropore volume on unmodified silicalite-1, whereas the lowest drop (or even no change) was observed for catalysts obtained with supports modified with 1.0 M XOH. These results are in line with the loss of crystallinity of the catalysts compared to the supports, as estimated based on the FTIR data.

**Figure 6:**
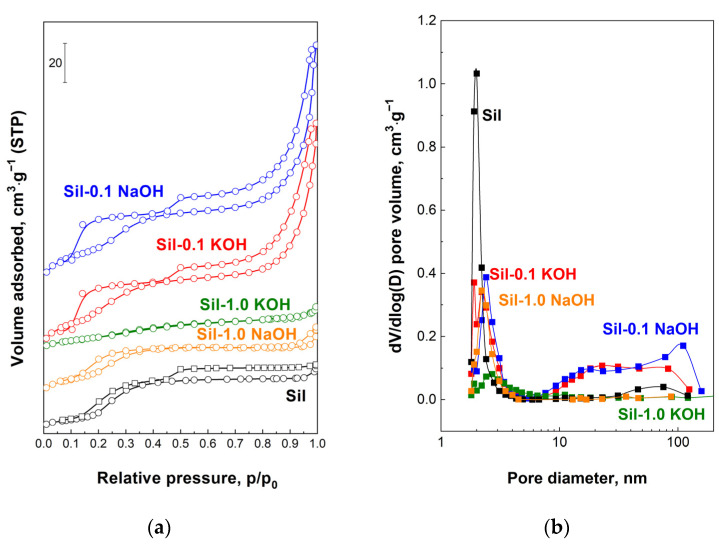
N_2_ adsorption/desorption isotherms (**a**) and pore size distribution (**b**) for indicated supports.

Figure 6 shows the N_2_ isotherms and the corresponding pore size distribution of the supports. The isotherms of the unmodified silicalite-1, as well as modified samples, exhibit two hysteresis loops. The first one at ~0.2 p/p_0_ occurs for silica MFI materials and its broadening for samples modified with 0.1 M XOH is a result of the increasing amount of defects formed during alkali modification [22,29]. Such a hysteresis loop does not change for a support modified with a 1.0 M NaOH solution. This is probably due to the low crystallinity of Sil-1.0 NaOH, and the formation of defects in the framework of this sample is balanced by its partial amorphization. In the case of Sil-1.0 KOH, amorphization predominates over defect formation, and, as a result, the low-pressure hysteresis loop disappeared. The second hysteresis loop (p/p_0_ > 0.45) for the initial sample is attributed to the intercrystalline porosity. The increasing volume of the second loop for the samples modified with 0.1 M XOH is associated with the generation of additional mesoporosity during the applied procedure. It is illustrated by the the pore distribution (Figure 6b) as the additional pores with larger diameters compared to the pores of the starting material. However, for supports modified with 1.0 M alkali solutions, the high-pressure loop disappears due to the connection of the silicalite-1 crystals into more dense, glued agglomerates during modification, as evidenced by SEM analysis (Figure 4d). The N_2_ isotherms and pore size distributions for the catalysts are similar to those of the supports.

**Figure 7:**
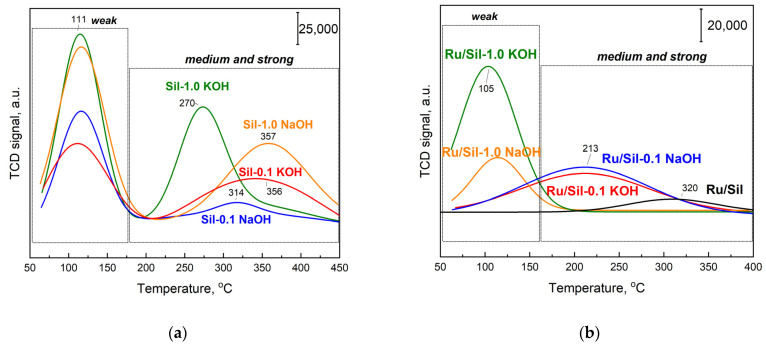
TPD-CO_2_ profiles for supports (**a**) and corresponding Ru/Sil catalysts pre-reduced in H_2_ at 500 °C (**b**); TPD-CO_2_ condition: saturation at 50 °C under CO_2_ flow for 60 min; heating at 10 °C/min under pure He flow.

To prove the effect of support modification on the nature of the basic sites, temperature-programmed desorption of CO_2_ (TPD-CO_2_) was conducted (Figure 7). TPD-CO_2_ analysis was performed for all of the calcined supports (Figure 7a) and the catalysts (Figure 7b) that were pre-reduced in situ under H_2_ at 500 °C before CO_2_ adsorption at 50 °C. Table 2 reports the corresponding amounts of desorbed CO_2_ calculated by integration of the relevant profiles and the density of basic sites. Only the initial, unmodified support (silicalite-1) did not show CO_2_ adsorption (straight line) and its content of basic centers was 0 (Table 2). After modification with alkali metal compounds, supports exhibited CO_2_ desorption dependent on the type and concentration of the modifier. Two CO_2_ desorption peaks with maxima at 110 °C and in the range of 200–450 °C are attributed to weak and medium/strong CO_2_ chemisorption, respectively, indicating the basic properties of the modified systems. Similarly, the presence of two types of centers were observed by Sun, et al. for Ru/CeO_2_ catalysts [30] and Gao et al. [31] for La_2_O_3_ -modified SiO_2_.

The introduction of the Ru active phase causes the decrease in the concentration of CO_2_ desorption centers for catalysts with modified supports (Figure 7b, Table 2) compared to the initial supports, whereas the concentration of centers able to adsorb CO_2_ for unmodified silicalite-1 increased after the introduction of the Ru phase. This suggests that the presence of ruthenium particles on unmodified Sil generates new centers able to adsorb CO_2_. The desorption maximum is present at a high temperature (~320 °C—Figure 7b), indicating the formation of strong adsorption centers. Similarly, Cobo et al. [32] studying modified 5% Ru/Al_2_O_3_ catalysts showed that CO_2_ is adsorbed not only on the basic centers present on the support surface, but also on Ru sites.

The decrease in CO_2_ adsorption concentration for Ru/Sil-1.0 NaOH and Ru/Sil-1.0 KOH catalysts is associated with the formation of NaCl and KCl, respectively, on their surfaces during the introduction of the active phase from RuCl_3_, as confirmed by XRD studies—Figure 3. In the case of Ru/Sil-1.0 NaOH and Ru/Sil-1.0 KOH catalysts, there is a noticeable disappearance of the medium/strong centers (in the range 200–450 °C—Figure 7b), suggesting that these centers are involved in the formation of the respective chlorides, while also participating in CO_2_ activation during methanation. The total amount of CO_2_ desorbed was used as a metric of basic density over the catalysts (Table 2). The Ru/Sil-1.0 KOH catalyst had the highest basic density, 0.23 µmol m^−2^, almost twice that of Ru/Sil-1.0 NaOH, 0.12 µmol m^−2^, and almost six times more than Ru/Sil-0.1 NaOH and Ru/Sil-0.1 KOH catalysts.

**Figure 8:**
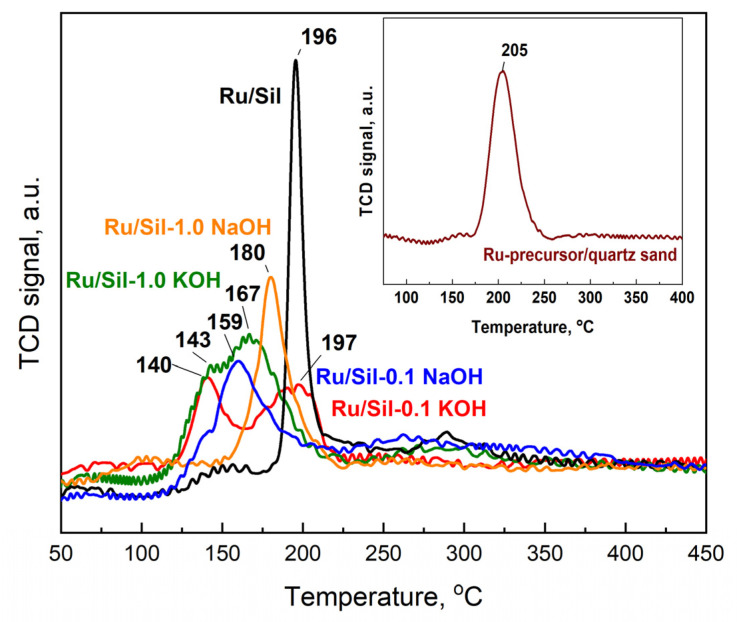
TPR-H_2_ profiles of the dried ruthenium catalysts and RuCl_3_·nH_2_O precursor supported on the quartz sand (inset). Signal intensity was normalized to 100 mg.

To obtain information regarding the reducibility of the active phase precursor, ruthenium chloride was deposited on the investigated supports and temperature-programmed reduction with hydrogen (TPR-H_2_) studies were performed. The TPR-H_2_ tests were carried out for dried catalysts, and their reduction profiles are presented in Figure 8. The initial analysis of the TPR profiles of the catalysts reveals that the maxima of the active phase precursor reduction occur at different temperatures for various supports. This indicates different strengths of interactions between the precursors and the surface of individual supports and/or different precursor dispersion. Quartz sand, on which the precursor—RuCl_3_·nH_2_O, was deposited using the impregnation method, served as a reference in the TPR studies.

The one-step reduction process has been recorded for the RuCl_3_·nH_2_O standard (Ru/quartz sand) and the Ru/Sil reference catalyst, with reduction peaks at 205 and 196 °C, respectively. The one-step reduction process of the RuCl_3_·nH_2_O precursor to Ru was recorded, among others, by the authors of the paper [33]. They showed that direct reduction of ruthenium chloride, without calcination, favors the formation of small crystallites of the active phase. The shift in reduction temperatures towards lower values for catalysts with modified supports relative to the standard and starting Ru/Sil is related to the better dispersion of the active phase on the support. This suggests that the modification of supports facilitates the dispersion of the active phase, resulting in smaller and more finely distributed crystallites of ruthenium, which can be beneficial for catalytic activity.

Moreover, the reduction process of RuCl_3_·nH_2_O for catalysts with modified supports occurs in two steps, which is most visible in the profiles of the Ru/Sil-0.1 KOH and Ru/Sil-1.0 KOH catalysts. The reduction peaks occurred at temperatures of 140 °C and within the range of 165–200 °C, respectively. In the case of catalysts modified with a NaOH solution, two clear reduction peaks were not observed, and only slight inflections appeared on the TPR-H_2_ profiles—Figure 8.

The two-step reduction corresponds to the reduction of surface Ru^4+^ species, formed during catalyst drying, to Ru^2+^ and then to Ru^0^, while the high-temperature analogue for the Ru/Sil catalyst corresponds to the direct reduction of the chloride precursor to metallic ruthenium. Similar reduction profiles of ruthenium catalysts were observed by the authors of [34,35] for a Ru/Al_2_O_3_ catalyst, Esen et al. [36] for Ru/SiO_2_ or Zieliński et al. [37] for Ru/MgF_2_ catalysts. The above studies indicate that the use of a modified system as a support of ruthenium facilitates its reduction process.

The TPR-H_2_ results have been correlated with those obtained from hydrogen chemisorption or TEM. The ruthenium dispersion of the samples was determined by H_2_ chemisorption considering a stoichiometry H/Ru = 1 [38]. From the dispersion values, an average particle size was estimated considering spherical particles. Blank experiments proved that the H_2_-pretreated initial and modified supports did not adsorb hydrogen. The results of hydrogen chemisorption measurements suggest that the textural and basic properties of the supports influence the dispersion and particle size of the active ruthenium phase.

The smallest particles were observed in Ru/Sil-0.1 NaOH and Ru/Sil-0.1 KOH, with particle sizes of 2.1 (D = 50%) and 2.9 nm (D = 35.2%), respectively (Table 3). The Ru/Sil catalyst exhibited the lowest dispersion (D < 5%). The highest dispersion shows the catalysts were supported on supports modified with low concentration of alkali solution. The dispersion of the active phase was found to be three times higher for Ru/Sil-0.1 NaOH than for Ru/Sil-1.0 NaOH and almost twice for the Ru/Sil-0.1 KOH catalyst compared to dispersion of Ru/Sil-1.0 KOH. Although Ru/Sil-1.0 NaOH and Ru/Sil-1.0 KOH catalysts showed higher basicity compared to systems with 0.1 M solution-modified supports, this modification did not lead to better dispersion. The strength of the basic centers might have influenced the dispersion, in addition to their concentration. The Ru/Sil-0.1 NaOH and Ru/Sil-0.1 KOH catalysts exhibited basic center strengths in the medium/strong range, while the Ru/Sil-1.0 NaOH and Ru/Sil-1.0 KOH systems were in the weak range.

When categorizing the studied systems into two groups, modified with 1.0 M and 0.1 M solutions, we observe a clear trend in basicity. For the Ru/Sil 1.0 XOH systems, the basicity decreases similarly to the dispersion of the active phase in the following order:

Ru/Sil-1.0 KOH > Ru/Sil-1.0 NaOH > Ru/Sil.

Similarly, for the Ru/Sil 0.1 XOH systems, we observe a decrease in basicity and Ru dispersion in the order of:

Ru/Sil-0.1 NaOH > Ru/Sil-0.1 KOH > Ru/Sil.

Another factor influencing dispersion was the specific surface area (SSA). The catalysts Ru/Sil-0.1 NaOH and Ru/Sil-0.1 KOH had the highest specific surface areas, while the catalysts with supports modified with high-concentration alkali solution possess much lower surface areas. This indicates that the mesoporous structure and large SSA of the modified supports significantly affect metal dispersion.

**Figure 9:**
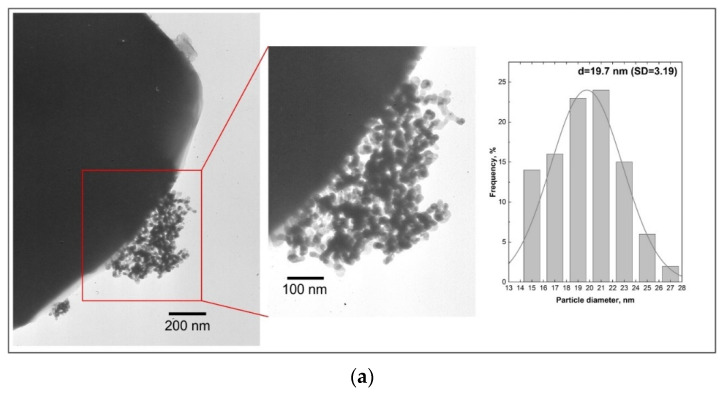

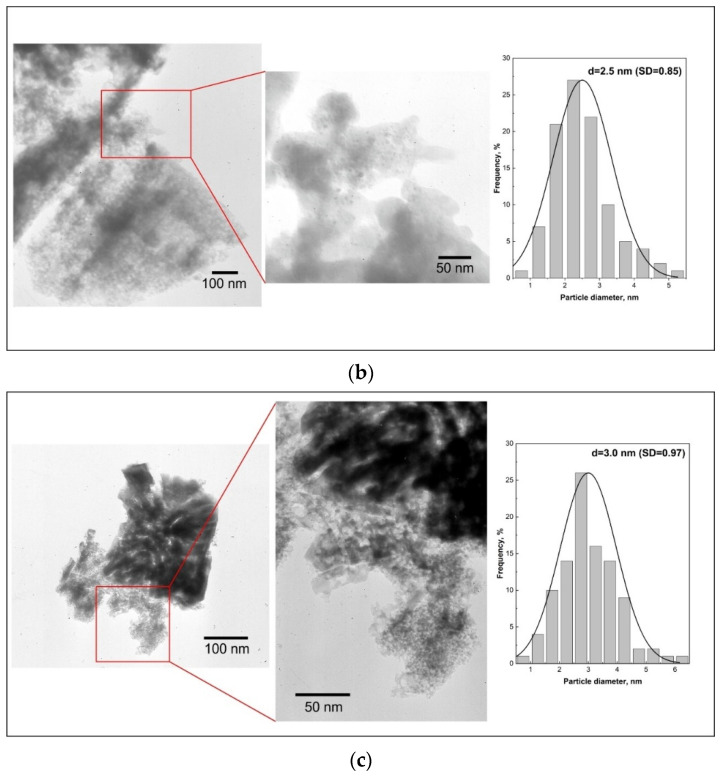
TEM micrographs of different magnitude and particle size distribution for the Ru/Sil (**a**), Ru/Sil-0.1 NaOH (**b**), and Ru/Sil-0.1 KOH (**c**) catalysts.

The changes in mean Ru particle size were characterized by TEM imaging. Representative images and the resulting particle size distributions (counted for at least 100 particles) are presented in Figure 9a–c for Ru/Sil (Figure 9a), Ru/Sil-01 NaOH (Figure 9b), and Ru/Sil-01 KOH (Figure 9c) catalysts, respectively. The darkest circular areas correspond to ruthenium particles due to the higher atomic number of Ru, with respect to other elements in the silicalite-1. Larger particles are observed for the Ru/Sil catalysts compared to the catalysts with the supports that present sodium or potassium in their composition. These results confirm the data obtained by chemisorption analysis.

**Figure 10:**
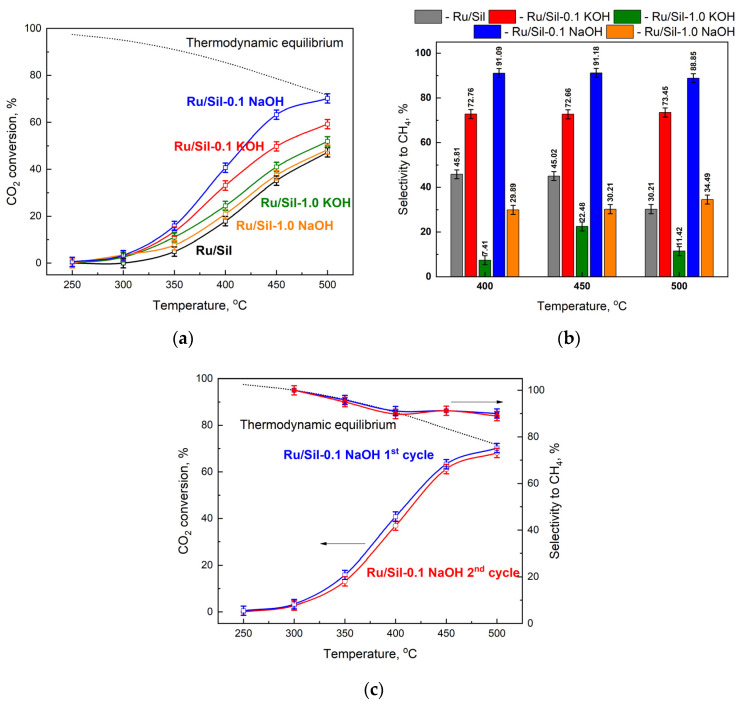
Catalytic activity tests: CO_2_ conversion (**a**) and CH_4_ selectivity (**b**) for the Ru catalysts, and catalytic stability of the Ru/Sil-0.1 NaOH catalyst (**c**). Reaction conditions: before reaction all catalysts (25 mg) were reduced in H_2_ at 500 °C, 30 min; the feed gas composition—H_2_:He:CO_2_ = 4:5:1; total flow rate—100 cm^3^·min^−1^.

The catalytic activity of the obtained catalysts was examined in the hydrogenation of CO_2_, and the comparison between the activity and the selectivity of ruthenium catalysts supported on unmodified and modified supports was discussed. The results of the catalytic tests performed at several temperatures in the presence of metals supported on different supports are presented in Figure 10a and the selectivity towards CH_4_ in Figure 10b. The supports (Sil, Sil-0.1 XOH or Sil 1.0 XOH; X-K or Na) did not show any activity in the methanation of CO_2_, while the reduced ruthenium catalysts were active in this process. Methane and CO were the only reaction products obtained over the tested catalysts.

Another important element of the response under study is the monitoring of carbon balance (CB). Carbon deposition theoretically does not occur if the H_2_/CO_2_ ratio is equal to or higher than the stoichiometric ratio [39]. Our studies were conducted at atmospheric pressure and at temperatures of up to 500 °C. Temperatures above 500 °C can cause sintering of Ru particles and increase carbon deposition, leading to catalyst deactivation. Thus, temperature control is vital as the exothermic methanation reaction can result in an apparent temperature increase in large-scale operations [40]. For this reason, the CB was monitored and was close to 1.0 at each temperature, meaning that there was no degradation of carbon dioxide.

The activity of all catalysts increases with increasing process temperature, reaching a maximum at 500 °C. Due to thermodynamic equilibrium, the reaction was not carried out at higher temperatures. The activity of the catalysts mainly depended on the type of support used. The lowest activities, over the whole temperature range, were observed for the Ru/Sil catalyst. The modification of silicalite-1 and its use as a support led to catalysts with activities higher than the starting Ru/Sil. The studied systems can be categorized into two groups depending on the concentration of the modifier. The use of supports modified with a 1.0 M solution of KOH or NaOH led to an increase in the activity of ruthenium catalysts, but the use of supports modified with lower concentration solutions (0.1 M) made it possible to obtain much more active catalysts. Among the systems tested, the Ru/Sil-0.1 NaOH catalyst showed the highest activity. For example, at 450 °C, its activity was 25% higher than that of Ru/Sil-0.1 KOH and almost twice as high as that of Ru/Sil-1.0 NaOH (Figure 10a).

A much greater effect of support modification was observed on selectivity toward methane—Figure 10b. While the starting catalyst (Ru/Sil) had a selectivity to CH_4_ of 50% at 400 and 450 °C, using the supports modified with 1.0 M solutions resulted in a significant reduction in selectivity, especially for the Ru/Sil-1.0 KOH catalyst. Its selectivity was only ~7 and ~22% at 400 and 450 °C, respectively. Much better results were obtained for catalysts supported on the support modified with 0.1 M solutions. Thus, the highest selectivity of >90% was characterized by the most active Ru/Sil-0.1 NaOH catalysts.

For the most promising sample, Ru/Sil-0.1 NaOH, two cycles of reaction were conducted, in order to verify whether the increase in conversion and yield with the temperature was due to the first activation of the fresh catalyst or it can be considered as characteristic behavior of the reacting system, and thus obtainable on the spent catalyst also. The results of this test are reported in Figure 10c. As can be seen, the first and second cycles reported almost the same trend in CO_2_ conversion and methane selectivity. This leads to two conclusions: firstly, the increase in the activity with the temperature rise is a characteristic trend of the system; secondly, no significative changes in the catalyst occur during the first cycle of reaction, thus demonstrating its stability, at least in the investigated reaction time.

**Figure 11:**
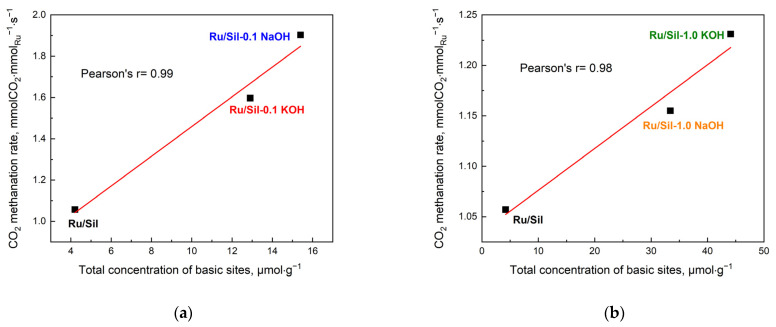
CO_2_ methanation rate as a function of content of basic sites for Ru/Sil 0.1 XOH (**a**) and Ru/Sil 1.0 XOH (**b**) catalysts (X = Na or K). Reaction conditions: before reaction all catalysts (25 mg) were reduced in H_2_ at 500 °C, 30 min; the feed gas composition—H_2_:He:CO_2_ = 4:5:1; total flow rate—100 cm^3^ min^−1^; T_reac_ = 450 °C.

**Figure 12:**
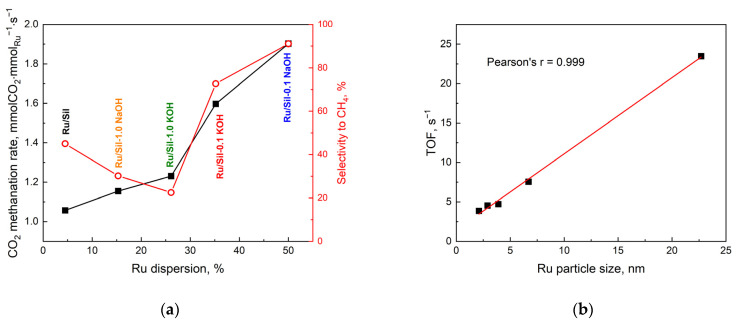
CO_2_ methanation rate and selectivity CO_2_ to CH_4_ as a function of dispersion for ruthenium catalysts at T_react._ = 450 °C (**a**) and the effect of Ru particle size on TOF (calculated by dividing the number of CO_2_ molecules converted per second by the number of active (surface) Ru atoms) (**b**).

The activity of the methanation catalysts was correlated with the concentration of the basic centers (Figure 11) and the dispersion and particle size of the active phase (Figure 12). This correlation is clearly visible in the case of our systems; an increase in total concentration of basic centers in the sample increases the total CO_2_ methanation rate—Figure 11a,b.

In addition, the activity of the catalysts tested is correlated with the dispersion of the active phase. Figure 12a shows the dependence of apparent rate (r_t_) and selectivity to CH_4_ on ruthenium dispersion. The catalyst with the best dispersion (Ru/Sil-0.1 NaOH) shows the highest activity. This seems to be a logical conclusion because the high dispersion of ruthenium provides a large number of active centers. Moreover, the same catalyst is also the most selective to methane. Thus, from a practical point of view, the catalyst with the highest dispersion is the right choice.

On the other hand, from a scientific point of view, it is interesting to study the intrinsic activity of metal crystallites of a specific size, which allows us to draw conclusions about the structural sensitivity of the CO_2_ methanation reaction. Figure 12b shows the dependence of CO_2_ methanation activity (expressed as TOF, s^−1^) on ruthenium particle size at 450 °C. The activity of the investigated catalysts increased with the particle size of the active phase—Figure 12b. This means that higher activity is shown by ruthenium centers located on large metal crystallites. However, the fact that there are far fewer of them (due to low dispersion) translates into lower overall activity. Similar correlations between turnover frequency of CO_2_ conversion and the size of crystallites were shown by the authors of a paper [41] studying the effect of Ni crystallite size on the activity of CO_2_ methanation.

A comparison of the CO_2_ methanation rate value obtained for the best Ru/Sil-0.1 NaOH catalyst (536 ${mmol}_{{CO}_{2}}$·g_Ru_^−1^·h^−1^; at 250 °C) with the results reported in the literature for ruthenium supported on Al_2_O_3_ (470 ${mmol}_{{CO}_{2}}$·g_Ru_^−1^·h^−1^) or MgO (320 ${mmol}_{{CO}_{2}}$·g_Ru_^−1^·h^−1^) [42], as well as ZrO_2_ (140 ${mmol}_{{CO}_{2}}$·g_Ru_^−1^·s^−1^) [43], shows that our ruthenium catalyst supported on modified silicalite-1 is a more promising catalyst.

## 3. Materials and Methods

### 3.1. Preparation of Supports

Silicalite-1 (denoted as Sil) was synthesized according to the procedure described in [22,44]. The synthesized silicalite-1 was calcined at 550 °C for 5 h in order to remove the template.

The calcined silicalite-1 was then modified with 0.1 M or 1.0 M solutions of alkali metal compounds (KOH (Stanlab) or NaOH (Chempur)). For this, 1 g of sample was mixed with 100 cm^3^ of the respective alkali hydroxide aqueous solution. The mixture was stirred under reflux at 60 °C for 1 h. Then, the samples were filtered, washed with deionized hot water, dried at 105 °C for 24 h, and then calcined in air for 3 h at 550 °C. The resulting samples were labelled as Sil-XY, where X stands for the molar concentration of the used solution, and Y stands for the used inorganic compound (e.g., Sil-0.1 KOH was prepared with 0.1 M KOH solution).

### 3.2. Preparation of Catalysts

Ruthenium catalysts were prepared by an incipient wetness impregnation method using RuCl_3_·nH_2_O (Aldrich, St. Louis, MO, USA) solution as a metal precursor. The Ru content in the catalysts was 1 wt.%. An appropriate amount of support was placed in an aqueous solution of RuCl_3_·nH_2_O followed by evaporation. After that, the supports with the metal precursor were dried at 105 °C for 24 h and reduced in a hydrogen flow for 2 h at 500 °C for characterization (BET specific surface area, FT-IR, XRD, H_2_ chemisorption, SEM, TEM, and TPD-CO_2_).

### 3.3. Characterization

The supports, as well as the catalysts, were characterized by means of different techniques. X-ray powder diffraction patterns (XRD) were collected on a Philips Bruker D8 Advance diffractometer using Cu Kɑ radiation (λ = 1.54056 Å) in the range of 2θ from 6° to 60°. FTIR spectra (KBr pellets) were obtained on a Bruker Tensor 27 spectrometer. The crystallinity (*C_IR_*) of the initial and modified supports, as well as catalysts, was determined based on the intensity of the bands characteristic for the MFI structure (at 550 and 450 cm^−1^) according to the formula [45]:

(5) CIR=I550I4500.72∗100%

Scanning electron microscope (SEM) images were recorded on the Hitachi SU3500 microscope. Transmission electron microscopy (TEM) images were recorded on a JEOL 2000 microscope operating at an accelerating voltage of 80 kV. N_2_ adsorption/desorption isotherms were measured at—196 °C using a Micromeritics ASAP 2010 sorptometer. Prior to the measurement, the samples were outgassed under a vacuum at 275 °C. The specific surface area was determined using the BET method, whereas the external surface area and micropore volume were calculated by the t-plot method. The total volume of pores was assessed using the single-point model (at p/p_0_ = 0.98). The BJH pore size distributions were derived from the adsorption branch. Ruthenium content in catalysts reduced at 500 °C for 2 h was determined by inductively coupled plasma optical emission spectroscopy (ICP-OES) on a Varian Vista-MPX spectrometer.

Measurements of temperature-programmed reduction with hydrogen (TPR-H_2_) and temperature-programmed desorption of carbon dioxide (TPD-CO_2_) were carried out on a Pulse ChemiSorb 2705 (Micromeritics) instrument. H_2_-TPR measurements were performed under a flow of 10 vol. % H_2_/Ar (20 cm^3^ min^−1^ (99.999%, Linde, Pullach im Isartal, Germany)) from 50 to 550 °C at a constant heating rate (10° min^−1^). The hydrogen consumption was monitored with a thermal conductivity detector (TCD) and the signal was normalized to the same sample weight of 100 mg. In the TPR-H_2_ studies, a quartz sand (Aldrich) impregnated with a ruthenium precursor was used as a reference material. The products of the reduction were retained by an isopropanol/liquid nitrogen cold trap at about −70 °C. Prior to the TPD-CO_2_ measurements, about 100 mg of the sample was pretreated in helium (He, 99.999, Aldrich) at 450 °C for 30 min, then cooled down to 50 °C and afterward saturated with carbon dioxide (CO_2_, 99.999, Aldrich) for 60 min. The physically adsorbed CO_2_ was removed by purging with a helium flow at 50 °C for 60 min and then the TPD analysis was carried out. All TPD-CO_2_ profiles presented in this work were collected in the temperature range of 50–450 °C with a heating rate of 10° min^−1^ and normalized to the same sample weight (100 g).

Hydrogen chemisorption measurements on supported metal catalysts were conducted by the static method at 100 °C on a Micromeritics ASAP 2010C sorptometer. Prior to hydrogen chemisorption, freshly dried catalysts were reduced with H_2_ (99.999%, Linde) at 500 °C for 30 min, and then the catalyst samples were pretreated in situ to purify their surfaces from adsorbed gases. The pretreatment consisted of evacuation at room temperature for 15 min. and then at 350 °C for 60 min., followed by a reduction in hydrogen flow (40 cm^3^·min^−1^) at 350 °C for 60 min. and evacuation at 350 °C for 120 min. Chemisorption of hydrogen was carried out at 100 °C and the isotherms were determined using 5 different pressures in the range of 50–310 mmHg. Assuming the stoichiometry of one hydrogen atom per one surface ruthenium atom (Ru_s_), the dispersion of ruthenium (D) can be expressed as D = Ru_s_/Ru_t_ = H/Ru_t_ (where Ru_t_–total number of ruthenium atoms in the sample).

Prior to the reaction of CO_2_ methanation, the samples were reduced with H_2_ at 500 °C for 30 min. The activity tests were performed with a feed stream containing CO_2_ and H_2_ in a stoichiometric ratio of 1:4, along with He in order to achieve 1:1 reactants:He dilution. Thus, the final feed stream ratio was H_2_:He:CO_2_ = 4:5:1 and the total flow rate was 100 cm^3^ min^−1^ (all gases—99.999%, Linde). The mass of catalyst used in these experiments was typically 25 mg. The catalytic performance for the CO_2_ hydrogenation reaction was evaluated using a fixed-bed reactor operating at atmospheric pressure in the temperature range of 250–500 °C. It should be noted that the reaction was carried out for 30 min at each temperature to achieve steady-state conditions. Gaseous reagents and products were measured every 50 °C with a chromatograph SRI Multiple Gas Analyzer #1 GC, equipped with a Silica Gel packed column, a Molecular Sieve 13X, and TCD detector, operating with He as the carrier gas. The CO_2_ conversion ($X_{{CO}_{2}}$) was calculated using the expression (6):

(6) $$X_{{CO}_{2}}=\frac{C_{CO,out}+C_{{CH}_{4},out}}{C_{{CO}_{2},out}+C_{CO,out}+C_{{CH}_{4},out}}*100$$

where *C_CO_*_,*out*_, $C_{{CO}_{2},out}$ and $C_{{CH}_{4},out}$ are the concentrations of CO, CO_2_, and CH_4_ in the outlet of the reactor.

Selectivities toward CH_4_ ($S_{{CH}_{4}}$) and CO (*S_CO_*) were calculated according to the expressions (7) and (8), respectively:

(7) $$S_{{CH}_{4}}=\frac{C_{{CH}_{4},out}}{C_{{CH}_{4},out}+C_{CO,out}}*100$$

(8) $$S_{CO}=\frac{C_{CO,out}}{C_{{CH}_{4},out}+C_{CO,out}}*100$$

where $C_{{CH}_{4},out}$ and *C_CO_*_,*out*_ are the outlet concentrations of CH_4_ and CO, respectively.

Carbon balance was calculated according to the expressions (9):

(9) $$Carbon\;balance\;(CB)=\frac{C_{{CH}_{4},out}+C_{{CO}_{2},out}+C_{CO,out}}{C_{{CO}_{2},in}}$$

where $C_{{CH}_{4},out}$, $C_{{CO}_{2},out}$, and *C_CO_*_,*out*_ are the outlet concentrations of CH_4_, CO_2_, and CO, respectively and $C_{{CO}_{2},in}$ is the concentrations of CO_2_ in the inlet the reactor.

## 4. Conclusions

In this study, we investigated the impact of basicity and textural properties of silicalite-1 modified with alkali compound (NaOH and KOH) supports on the activity of ruthenium catalysts in the hydrogenation of carbon dioxide to methane. We determined the effect of modifier type and concentration on the structure and basicity of the final supports. The use of 1.0 M solutions of KOH or NaOH enhances the basicity of the supports while diminishing their surface area. Conversely, employing a lower concentrated solution (0.1 M) leads to increased porosity of the supports and generates sufficient basicity for catalytic activity. The use of KOH solutions caused deeper changes of textural properties and basicity than the use of NaOH solutions. The use of supports with appropriate porosity and basicity positively affects the reducibility and dispersion of the active phase. Ru/Sil-0.1 KOH and Ru/Sil-0.1 NaOH catalysts exhibit easier reducibility, with the dispersion of the active phase being significantly better compared to the dispersion of catalysts obtained by using systems modified with 1.0 M solutions or the initial silicalite-1 (Ru/Sil).

The application of modified silicalite-1 as supports for the ruthenium phase allows for the creation of a new category of catalyst, characterized by high activities and selectivity in the hydrogenation of CO_2_ to CH_4_, surpassing those of the ruthenium system supported on unmodified silicalite-1. This activity correlates with the basicity and surface area of the catalyst supports, influencing not only the size of the ruthenium particles but also the efficiency of CO_2_ hydrogenation. Moreover, increasing the support’s basicity (Sil-1.0 NaOH or Sil-1.0 KOH) causes a shift in hydrogenation selectivity towards CO. The ruthenium catalyst supported on silicalite-1 modified with 0.1 M NaOH (Ru/Sil-0.1 NaOH) demonstrates excellent catalytic properties for the hydrogenation of CO_2_ to CH_4_.

The obtained results indicate that the combination of basic and structural properties of the supports enables the tailoring of selectivity towards desired reaction products. Ruthenium catalysts on modified silicalite-1 supports exhibit significant potential in carbon dioxide hydrogenation reactions.

## Figures and Tables

**Table 1:** Physicochemical characterization of supports and catalysts.

Sample Symbol	C_IR_^(a)^, %	S_BET_, m^2^·g^−1^	S_ext_, m^2^·g^−1^	V_tot_, cm^3^·g^−1^	V_micro_, cm^3^·g^−1^	V_meso_, cm^3^·g^−1^
Sil	100	315	82	0.19	0.12	0.07
Sil-0.1 NaOH	89	355	120	0.26	0.11	0.15
Sil-1.0 NaOH	73	299	112	0.16	0.09	0.07
Sil-0.1 KOH	83	348	164	0.26	0.09	0.17
Sil-1.0 KOH	66	181	40	0.10	0.07	0.03
Ru/Sil	86	318	111	0.16	0.06	0.10
Ru/Sil-0.1 NaOH	79	352	178	0.26	0.08	0.18
Ru/Sil-1.0 NaOH	71	290	115	0.17	0.09	0.08
Ru/Sil-0.1 KOH	74	336	186	0.24	0.07	0.17
Ru/Sil-1.0 KOH	59	202	68	0.11	0.06	0.05

^(a)^ C_IR_ crystallinity defined as (I_550_/I_450_)/0.72·100% (I_550_ and I_450_—the intensities of the bands at 550 and 450 cm^−1^)—see Experimental Section.

**Table 2:** Amounts of CO_2_ released during TPD-CO_2_ tests performed on calcined support and pre-reduced catalysts.

Sample Symbol	Total Concentration of Basic Sites, µmol·g^−1^	Density of Basic Sites, µmol·m^−2^
Sil	0	0
Sil-0.1 NaOH	16.8	0.05
Sil-1.0 NaOH	46.3	0.15
Sil-0.1 KOH	14.4	0.04
Sil-1.0 KOH	51.6	0.28
Ru/Sil	4.2	0.01
Ru/Sil-0.1 NaOH	15.4	0.04
Ru/Sil-1.0 NaOH	33.4	0.12
Ru/Sil-0.1 KOH	12.9	0.04
Ru/Sil-1.0 KOH	44.1	0.23

**Table 3:** Hydrogen chemisorption and CO_2_ hydrogenation activity on ruthenium catalysts reduced at 500 °C.

Sample Symbol	Hydrogen Chemisorption Data for Ruthenium Catalysts ^(a)^					CO_2_ Methanation Rate at 450 °C ${\mathrm{mmol}}_{{CO}_{2}}$·mmol_Ru_^−1^·s^−1^
	Volume Adsorbed, cm^3^·g^−1^			Dispersion, %	Particle Size of Ru ^(b)^, nm	
	H_t_	H_irr_	H_r_	D_t_		
Ru/Sil	0.05	0.01	0.04	4.5	22.7	1.057
Ru/Sil-0.1 NaOH	0.55	0.11	0.44	50.0	2.1	1.903
Ru/Sil-1.0 NaOH	0.17	0.08	0.09	15.3	6.7	1.155
Ru/Sil-0.1 KOH	0.39	0.18	0.21	35.2	2.9	1.597
Ru/Sil-1.0 KOH	0.29	0.06	0.23	26.1	3.9	1.231

^(a)^ Dispersion and mean size of Ru particles (in nm) were determined by H_2_ chemisorption at 100 °C; H_t_—total adsorbed hydrogen; H_r_—reversibly adsorbed hydrogen; H_irr_—irreversibly adsorbed hydrogen; D_t_—dispersion calculated from total adsorbed hydrogen. ^(b)^ The mean size of metal particles calculated from the amount of total chemisorbed hydrogen.

## Data Availability

The data presented in this study are available on request from the corresponding author.
